# Uncovering periodontitis-associated markers through the aggregation of transcriptomics information from diverse sources

**DOI:** 10.3389/fgene.2024.1398582

**Published:** 2024-06-11

**Authors:** Chujun Peng, Jinhang Huang, Mingyue Li, Guanru Liu, Lingxian Liu, Jiechun Lin, Weijun Sun, Hongtao Liu, Yonghui Huang, Xin Chen

**Affiliations:** ^1^ School of Physics and Optoelectronic Engineering, Guangdong University of Technology, Guangzhou, China; ^2^ School of Automation, Guangdong University of Technology, Guangzhou, China

**Keywords:** periodontitis, biomarkers, network analysis, integration, public microarrary datasets

## Abstract

**Introduction:**

Periodontitis, a common chronic inflammatory disease, significantly impacted oral health. To provide novel biological indicators for the diagnosis and treatment of periodontitis, we analyzed public microarray datasets to identify biomarkers associated with periodontitis.

**Method:**

The Gene Expression Omnibus (GEO) datasets GSE16134 and GSE106090 were downloaded. We performed differential analysis and robust rank aggregation (RRA) to obtain a list of differential genes. To obtain the core modules and core genes related to periodontitis, we evaluated differential genes through enrichment analysis, correlation analysis, protein-protein interaction (PPI) network and competing endogenous RNA (ceRNA) network analysis. Potential biomarkers for periodontitis were identified through comparative analysis of dual networks (PPI network and ceRNA network). PPI network analysis was performed in STRING. The ceRNA network consisted of RRA differentially expressed messenger RNAs (RRA_DEmRNAs) and RRA differentially expressed long non-coding RNAs (RRA_DElncRNAs), which regulated each other’s expression by sharing microRNA (miRNA) target sites.

**Results:**

RRA_DEmRNAs were significantly enriched in inflammation-related biological processes, osteoblast differentiation, inflammatory response pathways and immunomodulatory pathways. Comparing the core ceRNA module and the core PPI module, C1QA, CENPK, CENPU and BST2 were found to be the common genes of the two core modules, and C1QA was highly correlated with inflammatory functionality. C1QA and BST2 were significantly enriched in immune-regulatory pathways. Meanwhile, LINC01133 played a significant role in regulating the expression of the core genes during the pathogenesis of periodontitis.

**Conclusion:**

The identified biomarkers C1QA, CENPK, CENPU, BST2 and LINC01133 provided valuable insight into periodontitis pathology.

## 1 Significance

The development of periodontitis involves multiple biological processes. If not treated promptly, it will have a significant impact on an individual’s oral health, causing aggressive periodontitis, which leads to the destruction of periodontal tissues (Carvalho et al., 2018). Hence, we present a bioinformatics-based methodology for the identification of markers associated with periodontitis. This approach entails integrating diverse transcriptomic datasets and conducting a comparative network analysis to delineate functional pathways related to potential markers and differentially expressed genes in periodontitis. Through this methodology, we aim to elucidate the underlying mechanisms of periodontitis and unearth novel biomarkers. These findings are anticipated to contribute to the development of more precise strategies for early diagnosis, treatment, and prevention of periodontitis.

## 2 Introduction

Periodontitis is a common oral disease characterized by an inflammatory response of the periodontal tissues. The development and progression of periodontitis involve several biological processes, including bacterial infection, inflammatory response, immune regulation and tissue repair. If periodontitis is left untreated, it can ultimately result in tooth loss, significantly impacting an individual’s oral health and overall quality of life. Currently, diagnosing periodontitis relies on methods such as oral examinations, gingival probing and X-rays. Nevertheless, these methods have some limitations. In recent years, transcriptomics has become an essential tool for studying complex diseases (Supplitt et al., 2021). By analyzing the expression of genes, transcriptomics can reveal the molecular mechanisms and biological processes of diseases. For the study of periodontitis, transcriptomics can provide insight into the molecular basis of disease onset and progression (Liu et al., 2022a). However, transcriptomics research also faces some challenges (Jiang et al., 2015). Initially, periodontitis is a complex disease involving multiple biological processes (Cecoro et al., 2020). Transcriptomics data from a single source may not fully reflect the complexity of periodontitis. Secondly, transcriptomics data have high-dimensional characteristics, which poses challenges for data processing and analysis. Therefore, when studying the gene expression patterns related to periodontitis, appropriate methods must be employed to tackle and analyze these high-dimensional data. For instance, one may employ a differential analysis to discern variations in gene expression levels between healthy tissues and those afflicted by periodontitis.

Recently, scientists have initiated utilizing bioinformatics methodologies to discern and characterize biomarkers, striving to enhance their proficiency in recognizing disease-specific biomarkers. For instance, Ji et al. reveal the progression of human osteoarthritis with the help of single-cell RNA-seq analysis, integrating transcriptomics data from multiple biological specimens, such as blood, tissues and cell lines (Ji et al., 2019). Liu et al. identify osteosarcoma metastasis-associated signaling pathways with the help of logistic regression analysis (Liu et al., 2018). Yang et al. explored the relationship between MitoEVs and the immune microenvironment in periodontitis by using machine learning and bioinformatics methods (Yang et al., 2024). Cai et al. found a common pattern of gene expression between obesity and periodontitis by analyzing transcriptomic data and identified five important biomarkers (Cai et al., 2023). Huang et al. used machine learning combined with L1 regularisation and the LIME model interpreter to identify genes associated with periodontitis (Huang et al., 2023). He et al. analyzed the data for differential expression by bioinformatics techniques and screened for lncRNAs associated with periodontitis (He et al., 2023). Liu et al. used quantitative TMT proteomics and transcriptomics analyses to determine the protein expression profiles of patients with periodontitis and constructed nine representative biomarkers using machine learning models (Liu et al., 2022b). This approach can obtain more comprehensive and consistent information, improving the diagnosis and prediction of diseases. However, few studies have reported cases of using integrated multi-source transcriptomics data to identify markers related to oral diseases. For example, Wang et al. identify key markers of gingival tissue and immune cell infiltration studies in periodontitis (Wang Zihui et al., 2021). However, there may be batch effects and inconsistencies in data from different sources. It is necessary to consider some algorithms to remove the batch effects. Therefore, the introduction of RRA analysis in our study contributed to the further removal of batch effects Liu et al. use existing methods to analyze transcriptomics data from multiple sources, so it is possible to identify differential genes associated with pulpitis (Liu et al., 2021). Their approach took into account the batch effects from different sources of transcriptomic data. Therefore, We proposed methods for integrating multi-source transcriptome data to identify periodontitis-related markers by referring to the approach of Liu et al. Meanwhile, we refined the core gene identification process through a network comparative analysis involving both the core PPI network and the ceRNA network. The amalgamation of information from diverse networks contributed to the heightened accuracy and robustness of the identified biomarkers. Besides, we supplemented ceRNA network analysis based on their experiments. Functional enrichment analysis was performed in the core ceRNA network, which helped to uncover the major biological processes and pathways involved in the core ceRNA network. We performed correlation analysis of adjacent genes of core genes and identified lncRNAs with important regulatory roles.

## 3 Materials and methods

### 3.1 Workflow of the study

**FIGURE 1:**
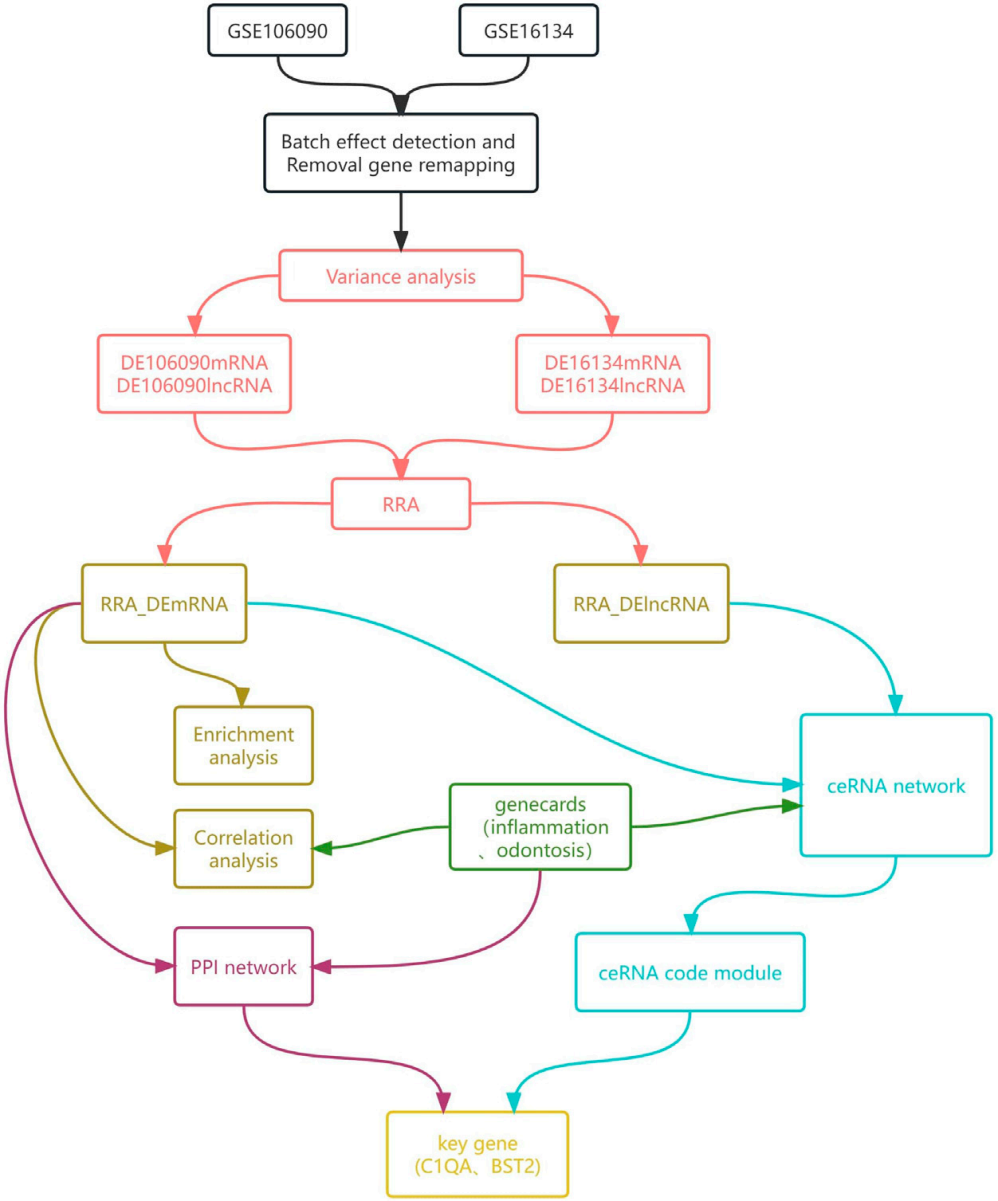
Flowchart of the study. The black part represents data preprocessing, the red part represents the integration and identification of differential genes, the brown part represents the processing of post-polymerization differential mRNAs, the green part represents the identification of inflammatory and odontogenic genes, the blue part represents the construction and analysis of the ceRNA network, the purple part represents the identification and analysis of the PPI network, and the yellow part is the final identification of the potential genes related to the mechanisms of periodontitis genesis.

This study summarized the workflow diagram in Figure 1.

Transcriptome datasets (GSE16134 and GSE106090) were collected from GEO, including proximal gingiva, distal gingiva and medial gingiva. The preprocessing included steps such as batch effect detection and probe remapping, which ensured data standardization between different datasets to eliminate differences due to batch effects. Then, the preprocessed data were subjected to differential analysis. The ranked list of differential genes (*p* < 0.05) was aggregated using RobustRankAggreg (version 0.6.1) to mitigate potential effects from smaller datasets. By employing an aggregation *p*-value threshold of less than 0.05 and aggregation ranking, we identified a list of differently expressed genes. Additionally, the differential mRNAs were subjected to functional enrichment analysis to understand their functions. To validate the correlation between the periodontitis genes and specific genes, we extracted inflammation-related genes and odontogenesis-related genes from geneCards. Subsequently, utilizing pre-existing miRNA targeting information, we constructed a ceRNA network comprising differential mRNA and lncRNA. MCODE was utilized to identify the core ceRNA module. Enrichment analysis was performed on the core ceRNA module to determine its associated functional pathways. Then we constructed a PPI network utilizing STRING and identified the core module of this PPI network by employing the MCODE plug-in. The core ceRNA module was compared with the core PPI module to identify potential genes implicated in periodontitis. Subsequently, we undertook a more detailed analysis of the correlation between the potential genes and associated genes within the core ceRNA network to pinpoint lncRNAs that played a crucial regulatory role in periodontitis.

### 3.2 Data processing

Unless specified otherwise, all data processing and statistical analyses were conducted using the R programming environment (R version 4.2.2). Gene expression profiling of GSE106090 involved screening the expression of lncRNAs and mRNAs in gingival tissues from six patients with peri-implantitis, six patients with periodontitis and six healthy individuals. GSE16134 was obtained from 120 systemically healthy periodontitis patients. The entire samples above were divided into diseased and healthy periodontal inclusion treatments. The collected transcriptomics datasets were tested for batch effects with the help of principal component analysis. The possible batch effects of GSE16134 and GSE106090 were removed from the batch effects with the help of the combat package (version 3.42.0).

### 3.3 Integration and identification of differential genes

Differential analysis was performed with the help of the limma package (version 3.48.3) and differential mRNAs and differential lncRNAs of the two datasets were extracted. Differential mRNAs and differential lncRNAs were obtained by threshold (*p* < 0.05, $\left|\log FC\right|\geq 0.3$ ) screening. The ranked gene list was subsequently aggregated using the RRA () function from the RRA package (version 0.6.1). Based on the amalgamated *p*-value (*p* < 0.05) and predefined significance threshold, a taxonomy of genes exhibiting differential expression had been determined.

### 3.4 Functional enrichment analysis

The differential gene sets were functionally annotated by the Gene Ontology (GO) and Kyoto Encyclopedia of Genes and Genomes (KEGG) analysis. The analysis of GO and KEGG was performed by using the clusterProfiler (version 3.18.0) package. The results of GO and KEGG enrichment analysis were subsequently visualized by the ggplot2 package (version 3.3.5) by plotting the bubble plot and the bar plot to show the enrichment of differential genes.

### 3.5 Correlation analysis

The expression data of differential genes after RRA analysis were extracted from the gene expression matrices of GSE16134 and GSE106090, and then the correlation analyses were performed respectively. The pearson method was chosen here to calculate the correlation coefficients. The correlation matrix was subsequently used to cluster genes with similar expression patterns together. The correlation coefficient matrix was visualized using heatmaps to show the correlation between different genes. Additionally, Inflammation-associated regulatory genes and odontogenesis-associated fundamental genes were discerned in geneCards, scrutinizing the correlation between these genes in both expression patterns.

### 3.6 Construction and scrutiny of ceRNA networks

The lists of differential mRNAs and differential lncRNAs were extracted from the results of differential analysis. Targets of miRNAs were derived from starBase (https://rnasysu.com/encori/). Based on the results of miRNA-mRNA prediction, relationships between differential mRNAs and miRNAs were screened. Additionally, in light of the miRNA-lncRNA target prediction outcomes, the interplay between the differently expressed lncRNAs and miRNAs was scrutinized. We ascertained mRNA-lncRNA pairs featuring identical miRNAs between differentially expressed mRNAs and differentially expressed lncRNAs. Furthermore, the mRNA-lncRNA pairs possessing overlapping miRNAs were deemed as nodes to construct a triangular relationship. Ultimately, we interlinked the triangular relationship between differentially expressed mRNAs and differentially expressed lncRNAs to form a comprehensive ceRNA network diagram. Subsequently, the ceRNA network identified in the preceding phase was imported into Cytoscape for comprehensive visualization of the ceRNA network. Afterward, the built-in plug-in NetworkAnalyzer of Cytoscape was used to calculate the metrics such as degree (number of connections) and centrality of each node (mRNA, lncRNA), and MCODE was used to screen the core ceRNA module. Subsequently, enrichment analysis was conducted on the pinpointed core ceRNA module to scrutinize their potential functional pathways.

### 3.7 Construction of PPI network

PPI network was constructed for differential mRNAs with the help of STRING’s built-in interaction relationships. Each gene represented a node, and their interaction relationship represented an edge. Additionally, the edges with a composite score ≥0.4 were selected for PPI network construction. For the constructed network, core module was selected by MCODE (parameters: degree cutoff: two, node score cutoff: 0.2, K-core: two) to identify tightly connected subgraphs in the network as gene modules with functional relevance. In PPI network analysis, Cytoscape’s built-in plug-in NetworkAnalyzer was used to calculate metrics such as degree (number of connections), centrality, etc., for each node (gene) to assess its importance in the network.

### 3.8 Correlation analysis of mRNAs and lncRNAs directly associated with core genes in the ceRNA core network

In the core ceRNA module, we selected those differential mRNAs and differential lncRNAs. Subsequently, core genes and their direct neighbor genes were extracted from gene expression matrices of GSE16134 and GSE106090. Subsequently, a correlation analysis was conducted. The pearson method was chosen to compute the correlation coefficient. Heat maps were utilized to project the correlation coefficient matrix, providing a visual display of the correlation between core genes and their direct neighbor genes.

## 4 Result

### 4.1 Differential mRNAs enriched in cell differentiation and regulation of toxic substances

**FIGURE 2:**
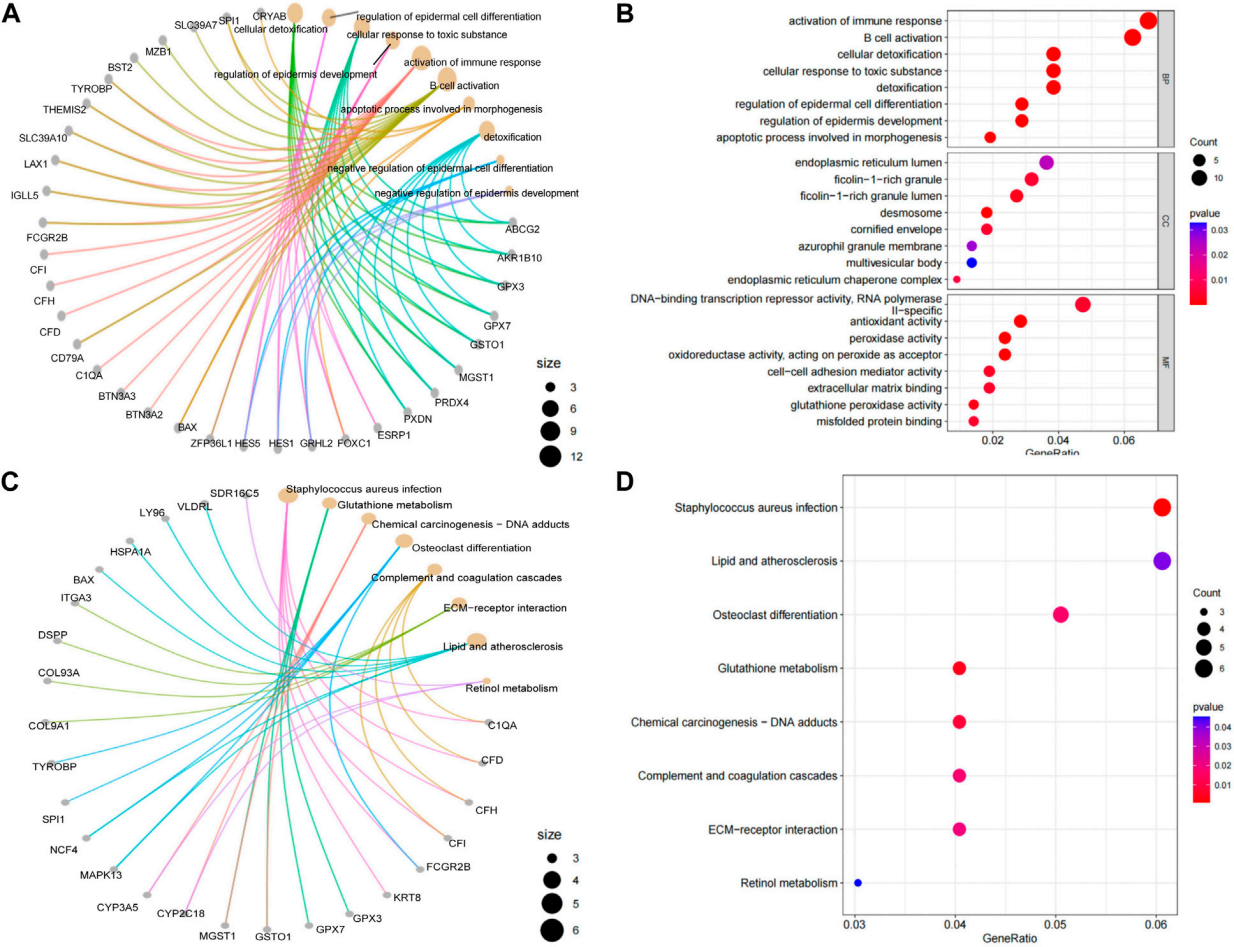
Results of GO/KEGG functional enrichment analysis of post-polymerisation differential mRNAs. Blueprint representation of **(A)** GO evaluation and **(C)** KEGG analysis via its network mapping. The distinctive mRNA GO/KEGG enrichment appraisal results are illustrated utilizing bubble charts and network maps, with the radius of the circles denoting the number of enriched genes. The tint of the circles mirrors the *p*-value, with all enriched terms deemed significant (*p* < 0.05). Network maps depict the distribution of genes residing within highly reinforced pathway clusters. **(B)** Enriched GO terms identified across the categories of biological processes, molecular functions, and cellular components. **(D)** Bubble chart of enriched pathway clusters identified through the KEGG pathway.

The detailed depiction of distinct gene integration and identification was illustrated in Table 1. The differential mRNAs and lncRNAs have been systematically identified based on the defined threshold (*p* < 0.05, $\left|\log FC\right|\geq 0.3$ ), encompassing a total of 670 downregulated differential mRNAs and lncRNAs, alongside 309 unregulated ones. After RRA analysis, a total of 13 unregulated mRNAs and lncRNAs were selected; 257 downregulated mRNAs and lncRNAs were identified. The enrichment analysis of GO and KEGG was performed on the selected differential mRNAs. Figure 2A summarised the top ten terms in the biological process (BP). Based on the results of GO enrichment analysis (Figure 2B), regulation of epidermal cell differentiation (GO:0045604, *p* = 4.58e-5) was the most significantly enriched BP in inflamed periodontium. Cellular response to toxic substances (GO:0097237, *p* = 6.55e-5) and regulation of epidermal development (GO:0045682, *p* = 8.66e-5) also had significant enrichment. KEGG pathway analysis (Figures 2C, D), osteoclast differentiation (hsa04380, *p* = 1.69e-2) was significantly enriched.

**TABLE 1:** The number of genes in differential analysis and RRA analysis.

Data	Differential analysis		RRA analysis	
Type	Up	Down	Up	Down
GSE16134mRNA	68	219	0	118
GSE16134lncRNA	23	1	13	0
GSE106090mRNA	184	407	0	126
GSE106090lncRNA	34	43	0	13

### 4.2 Periodontitis-related genes were highly correlated with inflammation and odontogenesis

**FIGURE 3:**
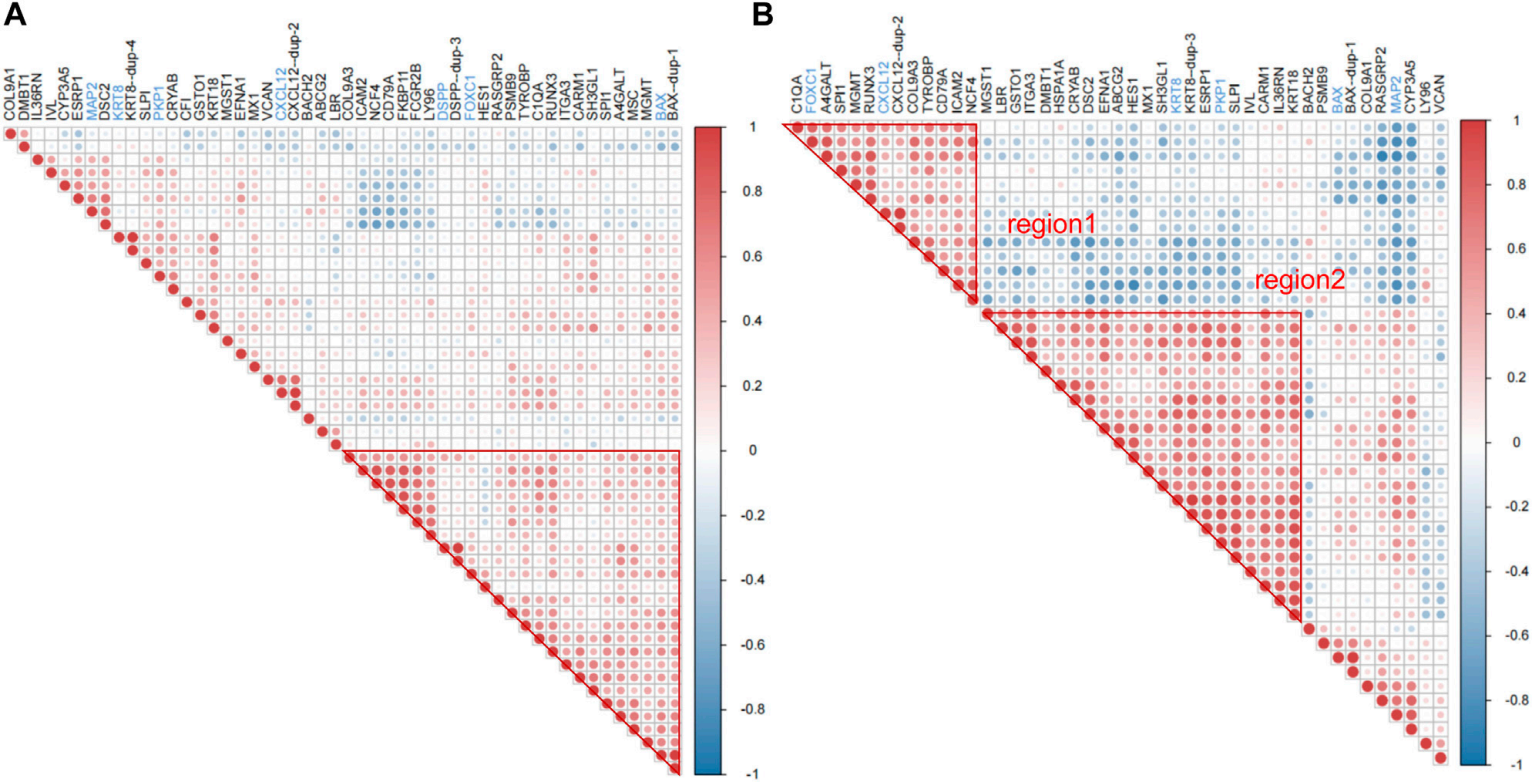
Results of correlation analysis of differential mRNAs for inflammation and odontogenic functions. We signifyed the extent of the positive or negative correlation through the color intensity (*p* < 0.05). Correlation coefficients exhibiting red or blue color respectively signify a positive or negative correlation. **(A)** The correlation analysis was conducted to examine the relationship between genes associated with odontogenesis and inflammation within the mRNAs that were differentially expressed in GSE16134. Among these, boxed in red are significantly associated with region. **(B)** The correlation analysis was conducted to examine the relationship between genes associated with odontogenesis and inflammation within the mRNAs that were differentially expressed in GSE106090. The top left corner red triangular area is designated as region 1. The central red large triangular area is designated as region 2. Red areas represent strong correlations, blue areas represent weak correlations, genes named in blackness are inflammation-related genes, and genes named in blue are odontogenesis-related genes.

Correlation analysis was a further validation and exploration of correlation between periodontitis genes. Based on the results of correlation analysis, it was known that in GSE16134 (Figure 3A), ICAM2, NCF4, CD79A, FKBP11, FCGR2B, LY96, DSPP, FOXC1, HES1, RASGRP2, PSMB9, TYROBP, C1QA, RUNX3, ITGA3, CARM1, SH3GL1, SPI1, A4GALT, MSC, MGMT and BAX had high correlation, among which DSPP, FOXC1 and BAX was associated with odontogenesis. In GSE106090 (Figure 3B), there were two highly correlated blocks, region1 for C1QA, FOXC1, A4GALT, SPI1, MGMT, RUNX3, CXCL12, COL9A3, TRYOBP, CD79A, ICAM2 and NCF4, region2 for MGST1, LBR, GSTO1, ITGA3, DMBT1, HSPA1A, CRYAB, DSC2, EFNA1, ABCG2, HES1, MX1, SH3GL1, KRT8, ESRP1, PKP1, SLPI, IVL, CARM1, IL36RN and KRT18. FOXC1, CXCL12, KRT8 and PKP1 were correlated with odontogenesis. Comparing the high correlation between the two data sets revealed that ABCG2, LBR, ITGA3, CARM1 and SH3GL1 had high correlation in both GSE16134 high correlation region and GSE106090 region2. C1QA, FOXC1, SPI1, MGMT, CXCL12, COL9A3, TYROBP, CD79A, ICAM2 and NCF4 were highly correlated in both GSE16134 and GSE106090 region1.

### 4.3 Inflammatory, odontogenic genes played hub gene roles in ceRNA networks

**FIGURE 4:**
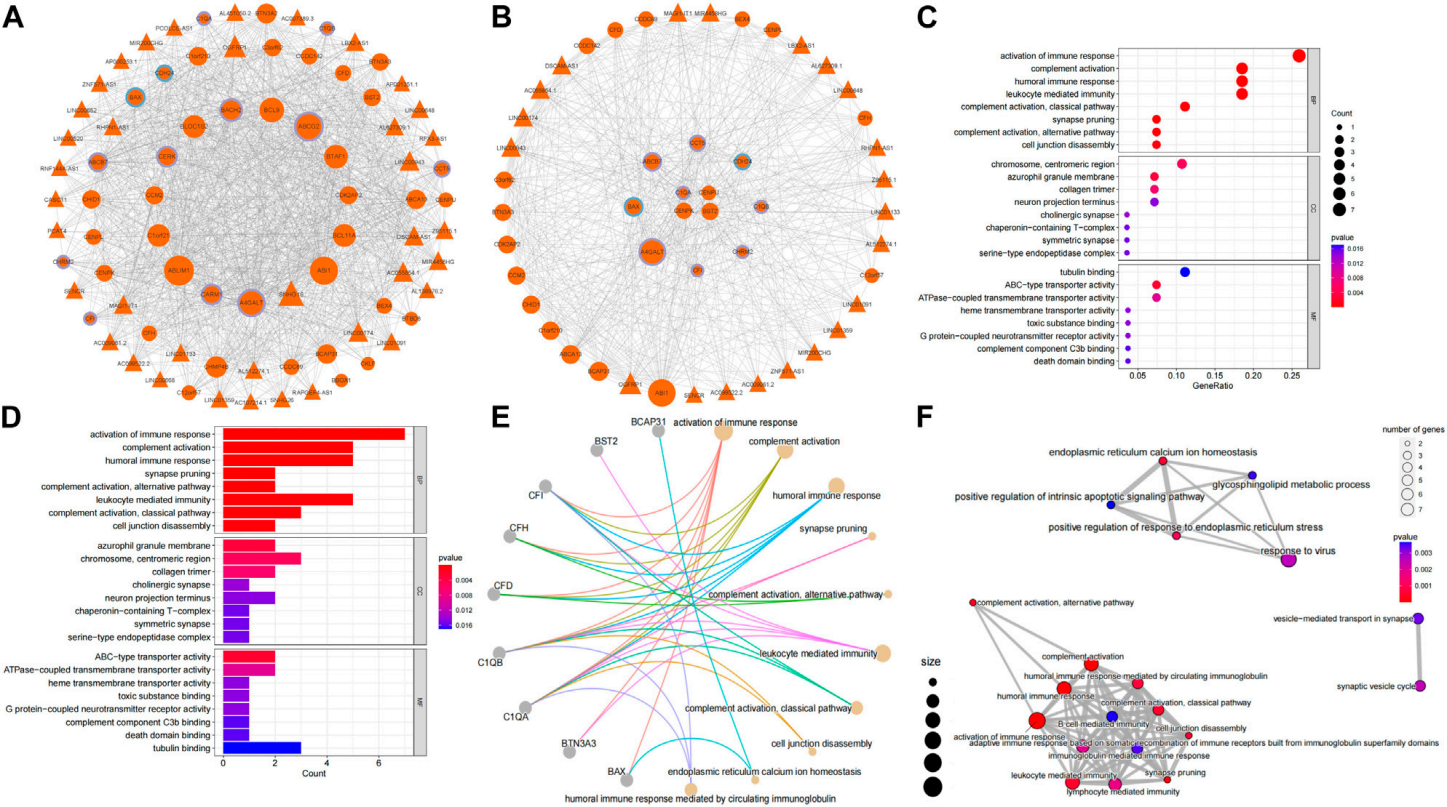
Construction of the ceRNA network and core network function mining results. **(A)** The ceRNA network: circles represent mRNAs, triangles represent lncRNAs, the size of the nodes indicates the degree of sparseness of connection with other nodes, node borders in purple indicate inflammation-related, blue indicates odontogenesis-related, and orange indicates that no correlation has been detected with both genes for the time being. **(B)** The ceRNA core module: node size and border settings refer to the ceRNA network. For the core ceRNA module, GO function enrichment analysis was performed for **(C)**, **(D)**, **(E)**, and **(F)**. Box plots and mesh plots were generated to visualize the result. GO functional enrichment analysis of core ceRNA modules, box plots, bubble plots, and mesh plots were drawn, and the settings of bubble plots and mesh plots were referred to in Figure 2. In the box plots, the longer the bands are, the more significant the functional enrichment is.

The correlation between RRA_DEmRNAs and RRA_DElncRNAs was selected by identical miRNAs to establish a ceRNA network, of which ten mRNAs were associated with inflammation, and two mRNAs were associated with odontogenesis (Figure 4A). The PPI network was extracted by the STRING platform (Supplementary Figure S1A), and core PPI module was identified by using the MCODE plug-in (Supplementary Figure S1B). They revealed a nucleus composed of C1QA, CENPK, CENPU and BST2 as central elements within the complex network. Additionally, C1QA exhibited a correlation with inflammation in geneCards, which implied that CENPK, CENPU and BST2 could feasibly manifest as factors linked to periodontitis. They potentially contributed to regulating the physiological processes associated with periodontitis. The core ceRNA module comprised 28 mRNAs and 21 lnRNAs (Figure 4B). Among these, seven mRNAs were found to be associated with inflammation, while two mRNAs were associated with odontogenesis. Among them, C1QA, CENPK, CENPU and BST2 were also core genes identified in the PPI network. Supplementary Figure S2 demonstrated that 11 lncRNAs were intrinsically linked to C1QA within the core ceRNA module. Additionally, 13 lncRNAs were directly linked to CENPU, and 17 lncRNAs were directly associated with CENPK. BST2 was directly related to 18 lncRNAs. Additionally, among the intersection of the lncRNAs linked to these core genes, there were six lncRNAs (LINC00943, LINC00174, DSCAM-AS1, MAGI1-IT1, MIR4458HG and LINC01133) that were universally linked to them. Notably, LINC00943, LINC00174, DSCAM-AS1 and MAGI1-IT1 were upregulated lncRNAs. Therefore, it was postulated that these six lncRNAs might play key roles in the regulation of gene expression in periodontitis.

### 4.4 Core ceRNA module enriched in immunoregulatory and complement activation

GO analysis of the core ceRNA module was performed to further reveal the biological significance of genes within the core ceRNA network. In the GO analysis, activation of immune response (Figures 4C, D) [GO:0002253, *p* = 1.06e-6, involving genes BAX/BTN3A3/C1QA/C1QB/CFD/CFH/CFI (Figure 4E)] was the most significantly enriched BP in the core ceRNA module. Complement activation (Figures 4C, D) [GO:0006956, *p* = 1.10e-6, involving genes C1QA/C1QB/CFD/CFH/CFI (Figure 4E)] and humoral immune response (Figures 4C, D) [GO:0006959, *p* = 8.01e-5, involving genes C1QA/C1QB/CFD/CFH/CFI (Figure 4E)] also significantly enriched. It can be seen that C1QA was significantly enriched in immunomodulation-related pathways. The top ten terms in the BP were summarized in Figure 4E.

### 4.5 LINC01133 played a role in regulating the expression of core genes in the pathogenesis of periodontitis

**FIGURE 5:**
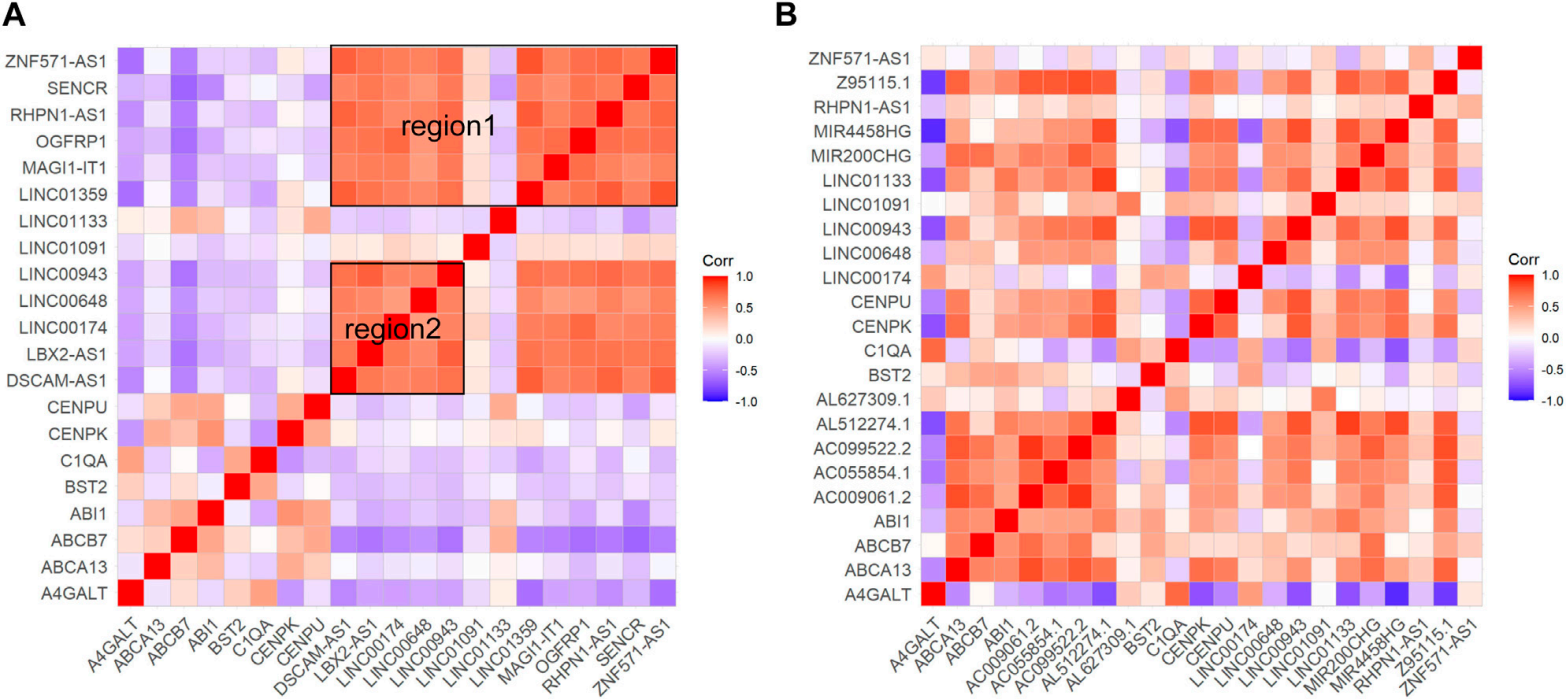
Results of correlation analyses of core gene-associated genes. Red hue signifies a positive correlation, whereas blue signals a negative one (*p* < 0.05). **(A)** Shows the correlation analysis of mRNAs and lncRNAs associated with the four core genes in the ceRNA core network in the GSE16134 expression profile, and **(B)** shows the correlation analysis of mRNAs and lncRNAs associated with the four core genes in the ceRNA core network in the GSE106090 expression profile. Among them, region 1 and region 2 within **(A)** have a high correlation.

The results of differential mRNA and lncRNA correlation analysis of direct association of core genes were performed in Figure 5. In GSE16134 expression profile analysis (Figure 5A), two regions appeared to have a high correlation of expression effect at either end of the diagonal with the upper right expression highly correlated. In the GSE106090 expression profile analysis (Figure 5B), most of the core-associated genes showed significant positive correlations. Then we compared with the higher correlation region in the GSE16134 expression profile, which revealed that C1QA and A4GALT, BST2; BST2 and A4GALT, ABCB7, C1QA; CENPU and ABCA13, ABCB7, ABI1, CENPK, LINC01133; CENPK and ABCA13, ABCB7, ABI1, CENPU, LINC01133 were positively correlated in both expression profiles. This observation strongly suggested that LINC01133 might serve as regulators in regulating the expressions of these core genes implicated in the development of periodontitis. Meanwhile, C1QA-BST2 and CENPU-CENPK were highly correlated with each other in the core genes. It can be hypothesized that CENPK, CENPU, BST2 and LINC01133 were potential periodontitis-associated genes.

## 5 Discussion

Transcriptomics data analysis has emerged as a promising approach for periodontitis research. It enables the identification of markers associated with periodontitis, providing valuable support for diagnosis and treatment. However, transcriptomics data analysis is challenging due to the high dimension, complexity and diversity of the data. Li et al. point out that although a variety of algorithms have been designed to integrate spatial and single-cell transcriptome data, there are significant differences in how these algorithms work and their scope of application (Li et al., 2022). Spatial transcriptome data are highly non-ideal, including features such as complex data structure, low signal-to-noise ratio, high sparsity, and uneven coverage, which pose challenges for in-depth analysis of the data and parsing of biological information (Li et al., 2024). Therefore, efficient algorithms are necessary for identification and analysis. Additionally, integrating and normalizing different transcriptomics data sources is crucial for subsequent research (Hauser et al., 2017).

Therefore, the paper proposes an integrated approach to identify periodontitis-related markers using transcriptomics data from multiple sources. This method efficiently processes large-scale transcriptomics data and employs advanced analysis algorithms to accurately identify these markers. It also enables the integration of periodontitis-related transcriptomics data from different sources. Furthermore, concerns regarding diagnostic techniques and their impact on analysis quality are addressed by checking for batch effects and ensuring sample homogeneity and between-group variability. Remapped probes retain updated annotation information from various platforms. Therefore, this paper presents a comprehensive approach to identify periodontitis-related markers using transcriptomic data from multiple sources. Meanwhile, considering batch effects for both datasets, we performed batch effect removal and introduce RRA analysis to enhance the robustness of results. In addition, core genes and potential markers were previously determined by network analysis for experimental single network determination. In this study, PPI network and ceRNA network are used to identify core genes and potential markers. This allows for more comprehensive biological information. Moreover, the accuracy and robustness of the identified biomarkers can be enhanced by integrating the information from different networks.

From the GO and KEGG analysis, it can be seen that differential mRNAs were involved in several processes related to inflammation and immunity. Among the GO enrichment pathways, immunomodulation, B-cell activation, cell detoxification, epidermal cell differentiation, developmental regulation and apoptosis showed a more significant enrichment. The medical literature provides a wealth of information on the molecular and immunological mechanisms by which T cells and B cells are involved in the pathogenesis of inflammatory diseases (Gonzales, 2000). Cell detoxification has been shown to be significantly enriched in periodontitis (Lehmann, 2020). Suzuki et al. indicate that periodontitis-related genes are significantly enriched in epidermal cell differentiation (Suzuki et al., 2019). Salmon et al. have suggested that developmental regulation is involved in the pathogenesis of periodontal disease (Salmon et al., 2017). Recent studies have demonstrated the involvement of ER stress in periodontal disease (Jiang et al., 2022). Therefore, we assessed the relevance and differential expression of the expressions of ER proteins and activation of immune response pathway in the GO-enriched pathway. The expressions of ER proteins and activation of immune response pathway have a strong correlation (Supplementary Figure S3A). As can be seen from the box plots of differential expression, the expressions of ER proteins and activation of immune response pathway were significantly different in both inflammatory and normal samples (Supplementary Figure S3B, Supplementary Figure S3C, Supplementary Figure S4D). In KEGG enrichment pathways, osteoblastic cell differentiation, glutathione metabolism, the tonicity and coagulation cascades and ECM-receptor interactions presented more enriched pathways. Comparison of the core PPI module and the core ceRNA module revealed that C1QA, BST2 and TYROBP in the PPI core module were significantly enriched in the immunomodulatory and B-cell activation pathways. Willems et al. clearly indicate that the C1Q family (C1QA) is associated with immunoregulatory pathways and autoimmune diseases (Willems, 2021). Alvarez et al. found that BST2 genes associated with antiviral defense, interferon signaling and Toll-like receptor signaling were significantly upregulated in the OPM of VEH/SIV (Alvarez et al., 2020). Huo et al. found that TYROBP rich in complement, inflammatory response, interferon γ response, and TNF-α signaling via NF-κB (Huo and Wang, 2021). The core ceRNA module genes C1QA, BAX, CFD, CFH and CFI were significantly enriched in the immune regulation pathway. Ma et al. noted that CFD + fibroblasts show high expression of chemokines similar to iCAF in some types of tumors (Ma et al., 2023). Duan et al. found that CFH and CFI were associated with immunity and characteristic reflection of periodontitis (Duan et al., 2023). BST2 was significantly enriched in the B-cell activation pathway. Moreover, according to geneCards, C1QA, TYROBP and CFI were found to be associated with inflammation, while BAX was associated with the process of odontogenesis. Additionally, the results of the correlation analysis indicated a strong correlation between C1QA and TYROBP within the two datasets. In summary, it can be inferred that changes in differential mRNA expression were closely associated with inflammation and immune-related biological processes. These findings may contribute to an in-depth understanding of the molecular mechanisms of inflammation and immune regulation.

LncRNAs play a pivotal role in periodontitis and there is growing evidence that lncRNAs have diagnostic value. Wu et al. found that inflammation in diabetes-associated periodontitis can be attenuated by activating the CTBP1-AS2/miR-155/SIRT1 axis (Ng et al., 2024). Xia et al. found that long-chain non-coding RNA PVT1 can be involved in pulpitis pathogenesis by regulating miR-128-3p (Xia et al., 2022). We constructed a ceRNA network aiming to discover the regulatory relationship between differential mRNAs and differential lncRNAs. Meanwhile, we found ten inflammation-associated genes and two odontogenesis-associated genes within the geneCards. Additionally, C1QA, CENPU, CENPK and BST2 were also present in the core PPI module, and C1QA was identified to be highly correlated with inflammatory genes within geneCards from the core ceRNA network. We also found 11 lncRNAs related to the regulation of C1QA, 13 lncRNAs linked to CENPU, 13 lncRNAs linked to CENPK, 17 lncRNAs linked to CENPK and 18 lncRNAs linked to BST2. Six lncRNAs were found to be co-linked to the above four genes, namely, LINC00943, LINC00174, DSCAM-AS1, MAGI1-IT1, MIR4458HG and LINC01133, suggesting that these six lncRNAs may play a role in the regulation of gene expression in periodontitis. Meng et al. found that LINC00943 could attenuate MPP + -induced neuronal injury through the RAB3IP axis in SK-N-SH cells (Meng et al., 2021). Su et al. found that LINC00174 could attenuate cardiac muscle injury through p53-mediated autophagy and apoptosis (Su et al., 2021). Maimeti et al. found that LINC00174 as an immune regulator may have a regulatory role in low-grade gliomas (Maimaiti et al., 2021). Ning et al. found that DSCAM-AS1 can accelerate cell value-addition and migration in osteosarcoma through GPRC5A signaling (Ning and Bai, 2021). Wang et al. found that MAGI1-IT1 has a regulatory role in controlling the value-addition of gastric cancer (Wang et al., 2021). Sun et al. found that LINC01133 also has a regulatory effect on the value-added of gastric cancer (Sun et al., 2022). Zeng et al. found that CENPK has the potential to serve as a predictive marker gene for clinical prognosis and personalized immunotherapy in cancer patients (Zeng et al., 2021). Zhou et al. found that CENPU was a key gene in the development of LUAD, closely associated with the infiltration of various immune cells (Zhou et al., 2021). Shan et al. found that BST2 contributes to the promotion of metastasis, invasion and proliferation of oral squamous cell carcinoma (Shan et al., 2023). Through the above core gene-associated lncRNAs, it has been shown that the above lncRNAs were related to the value-added and differentiation regulation of cancer cells, and LINC00174 is related to immune regulation. Meanwhile, in the enrichment analysis of the core ceRNA network, it was found that the immune-regulatory pathway was the most significant enriched pathway, and C1QA was one of the genes of the pathway. Therefore, we suspected that LINC00943, LINC00174, DSCAM-AS1, MAGI1-IT1, MIR4458HG and LINC01133 may have similar regulatory roles in the proliferation and differentiation of stromal and neutrophil cells as those in the proliferation and differentiation of cancer cells. They may also have a role in the process of immune regulation.

In the enrichment analysis of core ceRNA network, C1QA was significantly enriched in immune activation, complement activation, humoral immune response and leukocyte-mediated immune pathway. Hajishengallis et al. collation of an exposition of how to intervene in periodontal disease mechanisms using complement dependence (Hajishengallis et al., 2019). In this study (Supplementary Figure S4A), ficolin-1 and C1QA have some correlation. Supplementary Figure S4B, Supplementary Figure S4C and Supplementary Figure S4D pointed out that the two have significant differences. BST2 was significantly enriched in leukocyte-mediated immune pathway. The enriched pathways in the PPI network were also immune-regulation related, which suggested to a certain extent that C1QA played a certain role in immune regulation in periodontitis, and BST2 was a more likely potential periodontitis regulator. Meanwhile, CENPU and CENPK also existed in the core PPI network and were associated with cancer cell value-added and differentiation-related processes. Thus, it was speculated that they were also potential regulators in diagnosing periodontitis.

In the core ceRNA network, LINC01133 were identified as positively correlated with coregulators. It implied that LINC01133 may play a role in regulating coregulators' expression in cells. The positive correlation indicated that the expression levels of LINC01133 were consistent with the trend of change in the expression levels of regulatory genes. This may imply that LINC01133 may be involved in the regulatory network of core genes by interacting with core genes, which in turn affects the expression levels of core genes. This positive correlation may provide a new explanatory mechanism that LINC01133 may regulate the expression of core genes by sharing miRNA binding sites with core genes as ceRNAs. Differential lncRNAs increase the expression of core genes by adsorbing miRNAs and blocking the inhibitory effect of miRNAs on core genes. Therefore, the identification of some differential lncRNAs positively correlated with core factor correlations may help to further understand the role of these differential lncRNAs in regulating core factor expression and cellular functions.

In the PPI network, we chose to polymerize the post-differential mRNAs for the study of the interactions network, once again reducing the problem of heterogeneity present in the data from different platforms. The core PPI network was identified within geneCards as containing seven genes related to inflammation, in which C1QA, BST2, CENPU and CENPK were simultaneously present in the core ceRNA module. So it can be inferred that C1QA played an essential regulatory role in periodontitis. Jahanimoghadam et al. describe the interactions of common DEGs by constructing a protein interaction network, in which C1QA is one of the core ceRNA regulators. They find that IF135, MX1, SPI1 and IF144L associated with it are in the periodontitis core PPI network, and C1QB in the periodontitis core ceRNA network (Jahanimoghadam et al., 2022). Wang et al. construct the PPI network of differential genes when studying the changes in gene expression profiles in the dorsal horn of the spinal cord after sciatic nerve injury, and C1QA plays a more central role in this network. Meanwhile, C1QA has a strong interaction with TYROBP and C1QB, while TYROBP also plays a certain regulatory role (Wang et al., 2017). C1QA and C1QB are found to have a high interplay relationship in the bioinformatics analysis studies on the regulatory role of inflammatory genes in dwarfism diseases by Yuan et al. and vascular dementia molecules by Shu et al. (Yuan et al., 2021; Shu et al., 2022). In our study, C1QB exists in the core ceRNA network in periodontitis differential mRNAs and differential lncRNAs, which can be seen in the existing studies indicating that C1QB and C1QA have a strong reciprocal relationship. It can be speculated that there is also a potential reciprocal relationship between C1QB and C1QA in the regulation of periodontitis genes. BST2, CENPU and CENPK are the potential undiscovered genes that are related to the regulation of periodontitis.

Integrating transcriptomics data from multiple sources can reduce bias and error, improving data reliability. It enables the identification of more differentially expressed genes related to periodontitis, enhancing our understanding of its pathogenesis. It allows for comprehensive bioinformatics analysis, including gene function annotation, pathway analysis and protein interaction networks, deepening our understanding of periodontitis-related markers. At the same time, the deletion of the six dental implant samples did not affect the final conclusion. However, limitations exist due to potential differences in sample processing and sequencing platforms, which may affect data consistency.

In comparison with other studies, our research highlights four major points of distinction. Firstly, we consider that multi-source transcriptomic data may introduce batch effects due to factors such as laboratory and sequencing platform. Therefore, the detection and removal of batch effects are carried out before the differential analysis, in order to increase the consistency of the data and reduce the possibility of false positives, and to improve the accuracy of the differential analysis. Secondly, two-net control analysis is used to identify potential markers of periodontitis molecules to help obtain a more comprehensive understanding of gene regulatory networks. The accuracy and robustness of identifying biomarkers can be enhanced by integrating the information of the PPI network and the ceRNA network. Moreover, the functional enrichment analysis of the core ceRNA network is added, which compares with the enrichment analysis of differential genes, and find that the two are interlinked in immune regulation. It can also help us to further determine whether the genes in the core ceRNA network are involved in specific signaling pathways. This can help to further reveal the key pathways that may be affected in the development of periodontitis. Finally, we performed a correlation analysis of the core adjacent genes in the core ceRNA network and identify some of the genes highly positively associated with the core genes. Potential cooperative relationships and therapeutic targets may exist for highly positively correlated genes. This helps to provide a deeper understanding of the molecular mechanisms and disease development of periodontitis. Although there are limitations due to potential differences in sample processing and sequencing platforms, overall it does not affect the identification and identification of potential markers of periodontitis.

In conclusion, this study identified potential markers in the diagnostic process of periodontitis, and analyzed the functional pathways and interactions of the core modules. These results provided candidates for molecular diagnosis.

## Data Availability

The datasets presented in this study can be found in online repositories. The names of the repository/repositories and accession number(s) can be found in the article/Supplementary Material.

## Supplementary material

SUPPLEMENTARY FIGURE S1 PPI network construction and MCODE mining of core networks. **(A)** The PPI network: the PPI network was constructed for the mRNAs with differences after polymerization, the node size indicates the degree of the sparseness of the association with other nodes, the node border color is purple for inflammation, the border color is blue for odontogenesis, and the border color is orange for the time being, no association was found with the two types of physiological processes. **(B)** The PPI core network: Significant PPI modules delineated by MCODE. Node attributes such as color, dimension, and bordering styles are altered by the parameters stipulated for the PPI network.

SUPPLEMENTARY FIGURE S2 Results of the identification of genes directly associated with core genes. **(A)** Represents an inseparable gene cluster directly linked to C1QA, **(B)** signifies another such gene cluster linked to CENPU, while **(C)** denotes yet another about CENPK. Lastly, **(D)** indicates a distinct gene cluster directly related to BST2. Within this figure, circles signify mRNA molecules whereas triangles denote lncRNAs.

SUPPLEMENTARY FIGURE S3 The correlation assessment of the expressions of ER proteins and activation of immune response pathway. The correlation between the expressions of ER proteins and activation of immune response pathway in this study was assessed using normalized data of GSE16134 and GSE106090. **(A)** The scatter plot of correlation, the abscissa is the mean of expression of genes within the activation of immune response pathway in GSE16134 and GSE106090. The ordinate is the mean expression of genes within the ER pathway. R is the correlation coefficient while the P-values reflect the significance of the correlation. **(B)** “ai_ average” is the mean of expression of genes within the activation of immune response pathway in GSE16134 and GSE106090. “er_ average” is 16 the mean expression of genes within the ER pathway in GSE16134 and GSE106090. The expression of two pathways in the **(C)** normal samples and **(D)** inflammatory samples. The P-values were calculated by *t*-test.

SUPPLEMENTARY FIGURE S4 The correlation assessment of C1QA and ficolin-1. The assessment method was consistent with the Supplementary Figure S3. **(A)** The scatter plot of correlation with the abscissa representing the mean of expression of genes within the ficolin-1 in GSE16134 and GSE106090. The ordinate is the mean expression of C1QA in GSE16134 and GSE106090. **(B)** “f1_ average” is the mean of expression of genes within ficolin-1 in GSE16134 and GSE106090. “C1QA” is the expression of C1QA in GSE16134 and GSE106090. The expression of C1QA and ficolin-1 in **(C)** normal samples and **(D)** inflammatory samples.
