# Supplementary figures and images for: Uncovering periodontitis-associated markers through the aggregation of transcriptomics information from diverse sources
Source: Front Genet. 2024 Jun 11;15:1398582. doi: 10.3389/fgene.2024.1398582 (PMC11196414; doi:10.3389/fgene.2024.1398582)

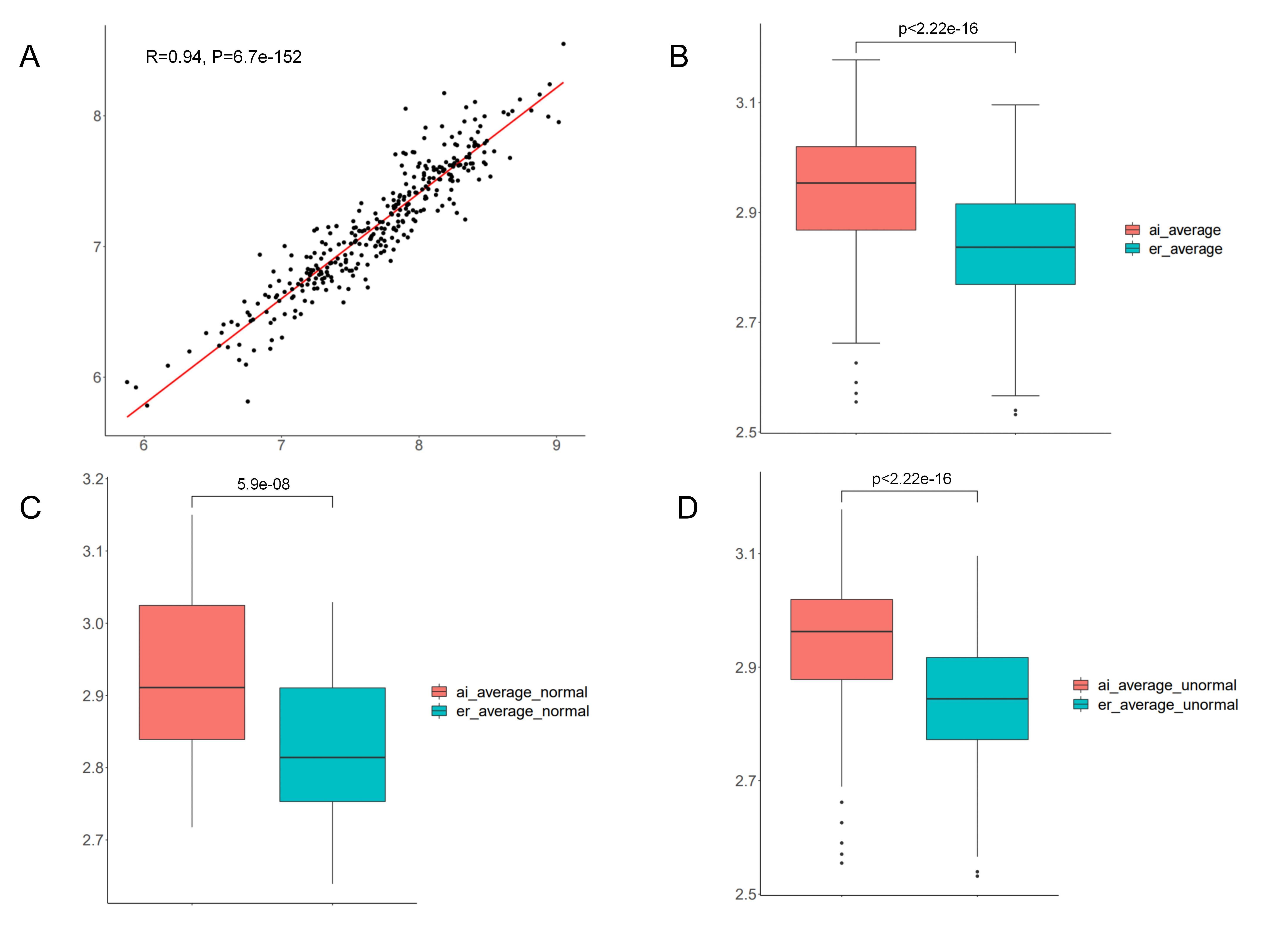

Supplement: Supplementary file 1 [file Image3.JPEG]

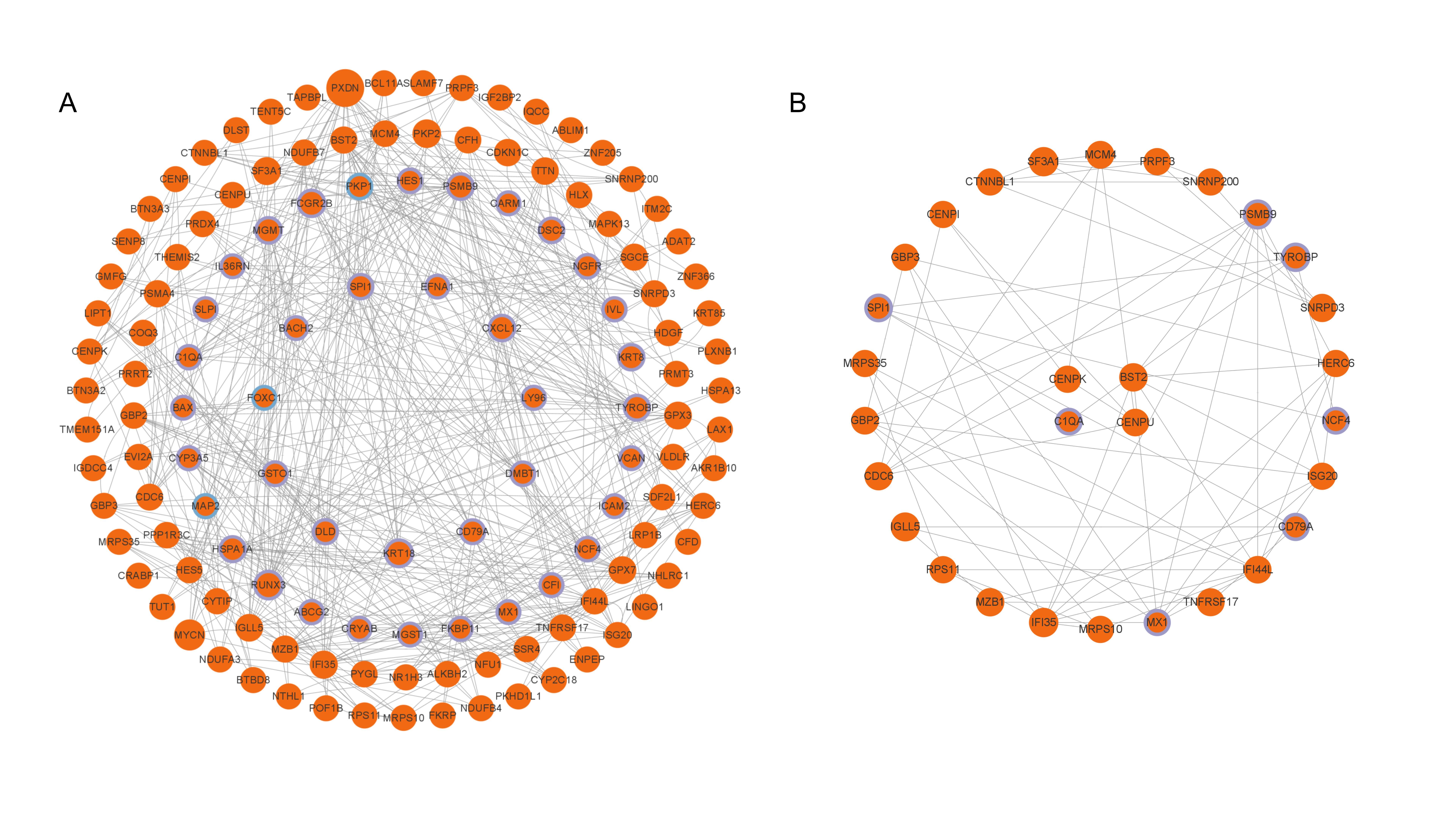

Supplement: Supplementary file 2 [file Image1.JPEG]

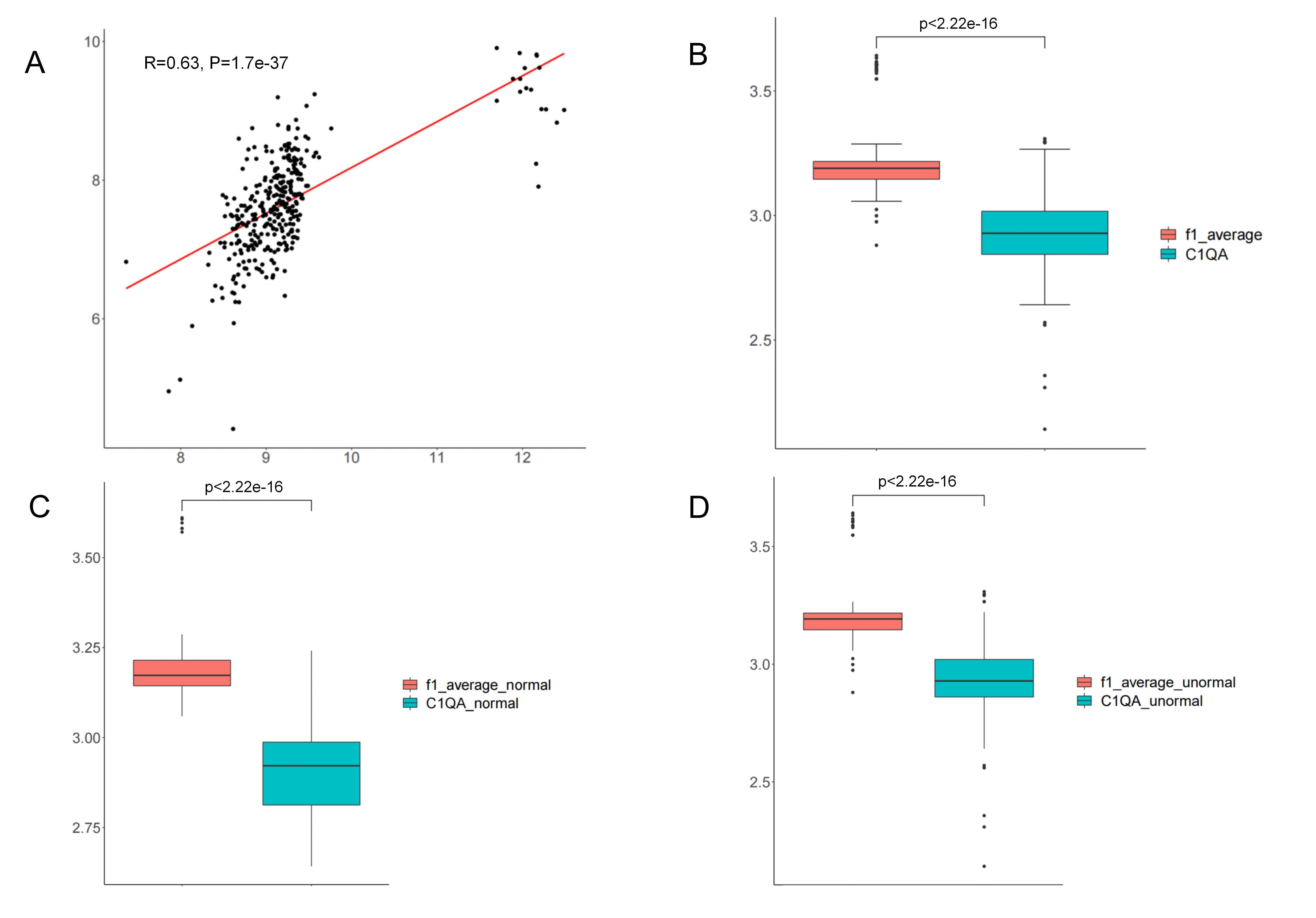

Supplement: Supplementary file 3 [file Image4.JPEG]

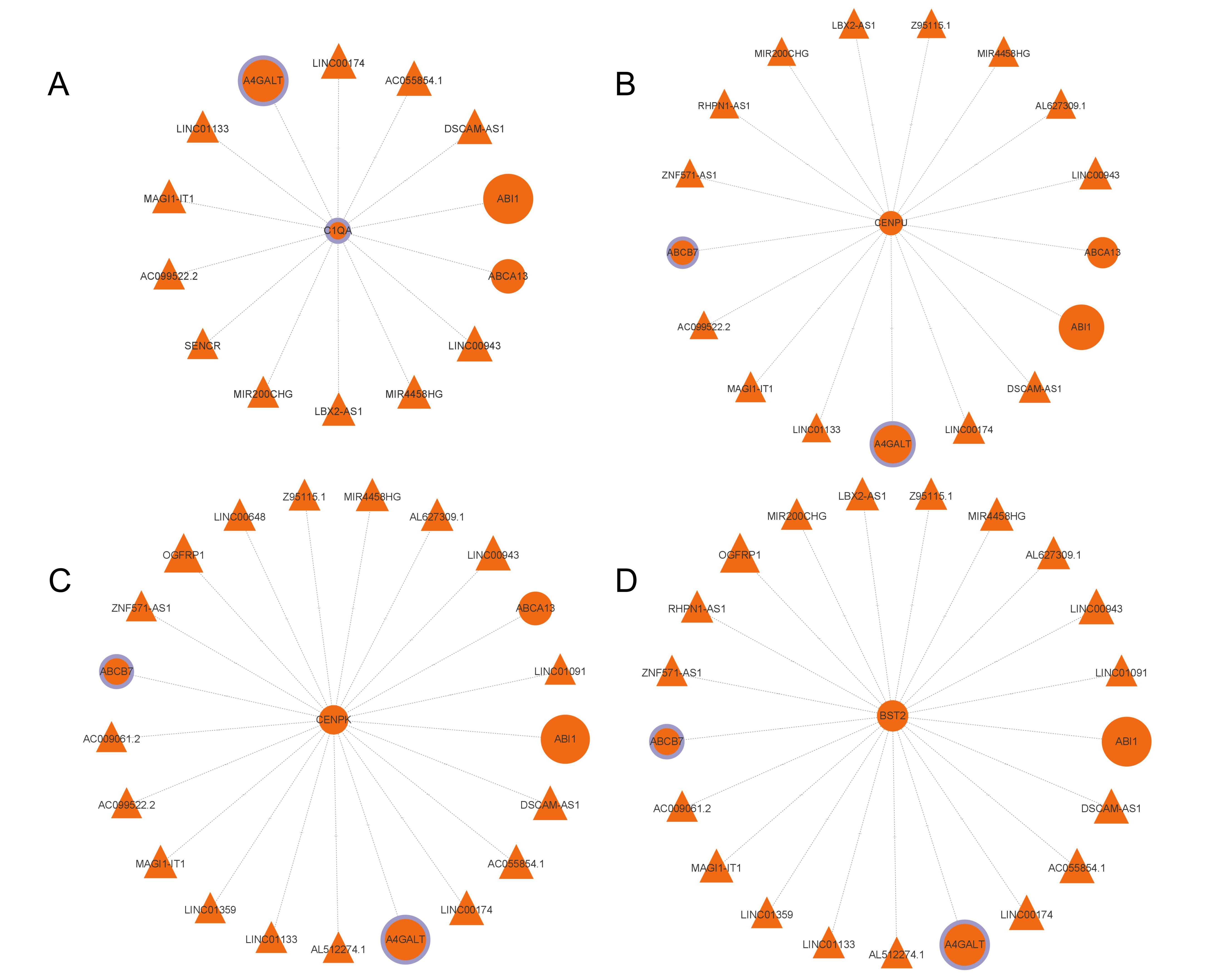

Supplement: Supplementary file 4 [file Image2.JPEG]
